# Cognitive and Histological Methodological Framework for an Intrahippocampal Aβ_1–42_ Rat Model of Alzheimer’s Disease †

**DOI:** 10.3390/neurolint18050079

**Published:** 2026-04-24

**Authors:** Loredana Mariana Agavriloaei, Bogdan Florin Iliescu, Gabriela Dumitrița Stanciu, Ivona Costachescu, Andrei Szilagyi, Maria-Raluca Gogu, Bogdan Ionel Tamba, Mihaela Dana Turliuc

**Affiliations:** 1 Department of Neurosurgery, Grigore T. Popa University of Medicine and Pharmacy Iasi, 700115 Iasi, Romania; 2 Department of Neurosurgery, “Prof. Dr. N. Oblu” Emergency Clinical Hospital, 700115 Iasi, Romania; 3 Advanced Research and Development Center for Experimental Medicine “Prof. Ostin C. Mungiu”—CEMEX, Grigore T. Popa University of Medicine and Pharmacy Iasi, 700115 Iasi, Romaniaandrei.szilagyi@umfiasi.ro (A.S.);

**Keywords:** Alzheimer’s disease, amyloid-β_42_, stereotaxic surgery, rat model, reduced cognitive performance, neurodegenerative

## Abstract

Background: Standardized and ethically compliant animal models remain essential for improving translational research in Alzheimer’s disease. Although Aβ_1–42_-induced rodent models are widely used, methodological variability continues to limit reproducibility. Methods: We explored the feasibility of a stereotactic intrahippocampal Aβ_1–42_ rat model established by bilaterally injecting pre-aggregated peptide into the hippocampus of adult Sprague Dawley rats. Model feasibility and targeting accuracy were assessed intraoperatively. Cognitive performance was evaluated using the Y-maze for spatial recognition memory and the novel object recognition (NOR) test. Histological examination was performed using hematoxylin–eosin (H&E) and Congo red staining to assess cytoarchitecture and to provide supportive evidence of amyloid-like deposits. Results: The surgical procedure was well-tolerated, and the injected animals showed reduced performance in behavioural testing, including reduced spatial recognition memory in the Y-maze and decreased discrimination indices in the NOR test. The animals also showed histological changes, including Congo red-positive birefringent structures consistent with amyloid-like congophilic material. Conclusions: This study presents a feasible experimental framework for intrahippocampal Aβ_1–42_ administration, showing behavioural and histological changes under the present experimental conditions. However, further validation, including sham-operated controls and molecular characterization, will be required before these findings can be interpreted as specific to Aβ-driven pathology.

## 1. Introduction

Alzheimer’s disease (AD) is one of the most devastating neurodegenerative disorders, representing 50–60% of dementia cases. Its global prevalence has increased markedly, with a 161% increase between 1990 and 2021, when it was last reported as a global burden [1]. In the United States of America (USA), there are 7.2 million people aged 65 years and older who live with AD [2]. A recent study reported that mortality related to Alzheimer’s disease has also seen a rise of 3.7% from 10.86% in 2012 to 13.71% in 2020 [3]. The 2025 Alzheimer’s disease report mentioned a 142.2% rise in deaths related to AD-related dementia in the American population between 2000 and 2022 [2]. Given these epidemiological trends, there has been growing interest in developing disease-modifying treatments. Despite substantial advances in understanding the pathophysiology of Alzheimer’s disease, the translation of preclinical findings into effective disease-modifying therapies remains limited. Several therapeutic strategies targeting amyloid pathology, including β-secretase inhibitors and anti-amyloid monoclonal antibodies, have demonstrated promising effects in experimental models but have yielded modest or inconsistent clinical benefits [4]. One of the factors contributing to this translational gap is the heterogeneity of preclinical models, including variability in amyloid preparation protocols, dosing strategies, stereotactic parameters, and outcome measures, which complicates reproducibility and limits comparability across studies [5]. 

Experimental models of Alzheimer’s disease are traditionally classified into transgenic models and intracerebral peptide-based models [6]. However, an increasingly important third category that has emerged comprises viral vector-mediated approaches, such as adeno-associated virus (AAV)-based overexpression of human APP, Aβ, or tau constructs (e.g., AAV-hTau, AAV-BRI2-Aβ42, AAV-APP). These models provide improved temporal control and regional specificity, thereby offering complementary advantages over classical approaches [7].

Among these, the stereotactic injection of pre-aggregated Aβ_1–42_ peptide into the rat hippocampus remains an accessible and reproducible approach. This approach provides control over the spatial and temporal aspects of peptide administration and local tissue exposure and is practical and cost-effective because of the larger hippocampus of rats compared with that of mice. Several reviews published to date describe considerable heterogeneity in amyloid dosing strategies, aggregation protocols, and assessed outcomes and consistently acknowledge the absence of a unified, systematic dose–response analysis [5,8]. Researchers use different protocols for fibril preparation, choose varying stereotaxic coordinates, and rely on their own distinct validation criteria [9]. These discrepancies continue to undermine reproducibility and make meaningful comparisons across studies quite challenging.

To better contextualize this heterogeneity, we provide a comparative overview of representative intrahippocampal Aβ_1–42_ protocols, summarized in Table 1. This variability may significantly influence both behavioural and histological outcomes and highlights the lack of methodological standardization in this field [10].

In this study, we aimed to establish a feasible and ethically compliant intrahippocampal Aβ_1–42_ administration framework in rats, integrating peptide preparation, stereotactic delivery, behavioural testing, and histological assessment within a structured experimental workflow. Cognitive function was evaluated using two low-burden, hippocampus-dependent tasks, the Y-maze spontaneous alternation and the Novel Object Recognition (NOR) test [20,21], paired with standard histological assessments, including hematoxylin–eosin (H&E) and Congo red staining [22].

## 2. Materials and Methods

### 2.1. Reagents and Instruments

Aβ_1–42_ peptide was purchased from MedChemExpress (MedChemExpress EU—MedChemTronica, Stockholm, Sweden). Aβ_1–42_ stock solution (1 mg/mL) was prepared in Phosphate-Buffered Saline (PBS: 0.01 M NaH_2_PO_4_, 0.15 M NaCl, pH 7.4) and stored at −20 °C. Before in vivo injection, aliquots of Aβ_1–42_ were incubated at 37 °C for 7 days to obtain insoluble fibrils of amyloid [23,24].

Aβ_1–42_ solution was analyzed to confirm amyloid fibril formation and assess ultrastructural morphology. The incubated solution was scanned with scanning transmission electron microscopy (STEM) using a Verios 64 UC microscope (Thermo Fisher Scientific, Hillsboro, OR, USA) [25].

### 2.2. Experimental Animals

The experimental protocol and procedures were conducted at the Advanced Research and Development Center for Experimental Medicine “Prof. Ostin C. Mungiu” (CEMEX) in compliance with the European Community Guidelines (Directive 2010/63/EU) and Romanian legislation (Law no. 43/2014) on the protection of animals used for scientific purposes. This experiment was ethically approved by the Ethical Committee at “Grigore T. Popa” University of Medicine and Pharmacy of Iasi (nr. 483/28.10.2024) and the Romanian National Sanitary Veterinary and Food Safety Authority (nr. 75/18.12.2024).

The animals were housed in individually ventilated cages (IVCs) within the animal facility at CEMEX. They were maintained under standard husbandry conditions, including a controlled room temperature (20 ± 4 °C), relative humidity (50 ± 5%), and a controlled light–dark cycle with unrestricted access to water and standard laboratory chow. Daily assessments were performed to monitor general health, feeding behaviour, and any signs of postoperative discomfort.

The animals were kept isolated in laboratory conditions for 7 days during the quarantine period and were monitored and clinically examined daily for possible disease or abnormal behaviour. The animals were purchased from the Cantacuzino National Institute of Research and Development for Microbiology and Immunology (Bucharest, Romania).

**Figure 1:**
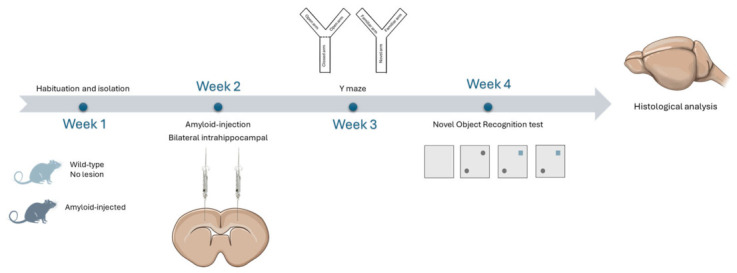
The schematic illustration of the experimental timeline spans 4 weeks. Week 1 involves the acquisition and acclimatization of animals. In week 2, animals undergo surgery with bilateral intracerebral administration of amyloid. In weeks 3 and 4, animals are subjected to the Y-maze and the NOR test, respectively. At the end of the protocol, brain tissue is collected for histological analysis.

In this methodological study, we used 12 rats, divided as follows: six in the wild-type (WT) group, which underwent no surgical intervention, and six in the amyloid-induced (AD) group. Animals were allocated to experimental groups according to the experimental design, without formal randomization. The AD group was subjected to the surgical procedure of amyloid injection in week 2. Both groups were tested cognitively in the Y-maze and with the NOR test. A schematic representation of the 4-week experiment is illustrated in Figure 1. Blinding was not systematically implemented across all experimental stages in this study.

Adult male Sprague Dawley rats were used. At the time of surgery, animals were 6 months old, corresponding to mature adult rats, with a body weight ranging from 350 to 400 g. Sprague Dawley rats were used because this strain has well-documented behavioural stability, docile temperament, and consistent physiological responses, which make it highly suitable for stereotaxic neurosurgical procedures and cognitive testing. Sprague Dawley rats also present a larger hippocampal volume compared to mice, facilitating accurate targeting and reproducible delivery of compounds during intracerebral injections [26]. Moreover, this strain is widely employed in neurodegenerative disease models, including amyloid- and tau-based paradigms, allowing for comparison with existing data and improving translational relevance [27].

### 2.3. Induction of Alzheimer’s Disease

The experimental model of Alzheimer’s disease was established through bilateral stereotactic injection of aggregated Aβ_1–42_ peptide into the hippocampus. The coordinates were selected according to the Paxinos and Watson rat brain atlas, plate 56: anteroposterior (AP) −2.76 mm, mediolateral (ML) ±2.0 mm, and dorsoventral (DV) −3.6 mm from the bregma.

**Figure 2:**
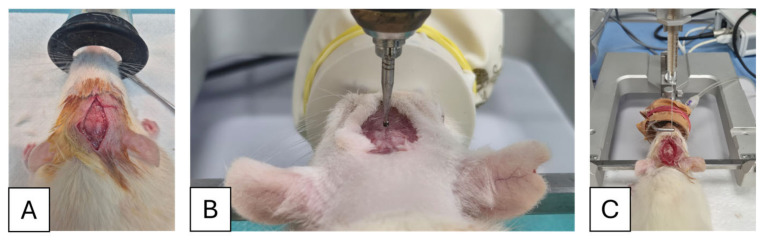
Illustration of the induction method. (**A**) After induction with 5% isoflurane, animals were placed on a heating pad with continuous 2.5% isoflurane flow through a mask, and an incision was made on the median line. (**B**) After skull exposure, the rats were placed in the stereotactic apparatus, and the coordinates (AP) −2.76 mm, mediolateral (ML) ±2.0 mm were set for bilateral drilling. (**C**) The drill was changed to the Hamilton syringe, and the coordinate for the hippocampus was set at −3.6 mm dorsoventral (DV).

Animals were anesthetized with isoflurane 5% for induction and 2.5% for maintenance while placed in a stereotaxic apparatus to ensure precise positioning. A Hamilton microsyringe, mounted on the injector arm, was used for bilateral delivery of the Aβ_1–42_ solution. Each hippocampus received 5 μL of Aβ_1–42_ solution (1 mg/mL), corresponding to a total dose of 5 μg per hippocampus, at a constant infusion rate of 1 μL per minute. Upon completion, the needle was left in situ for an additional 5 min to reduce reflux and facilitate adequate diffusion of the solution into the tissue. The needle was then withdrawn slowly, and the surgical site was closed with surgical glue (SURGIBOND^®,^ SMI AG, St. Vith, Belgium). Figure 2 illustrates the main steps in the induction protocol.

All procedures were conducted under aseptic conditions to minimize the risk of postoperative complications. Postoperative analgesia was provided using buprenorphine (0.01–0.05 mg/kg, subcutaneously), administered according to established protocols for stereotaxic procedures and repeated every 12 h for up to 3 days [13,28]. After recovery from anesthesia, animals were returned to their home cages and monitored until normal mobility was observed. Daily assessments were performed to monitor general health, feeding behaviour, and any signs of postoperative discomfort.

### 2.4. Cognitive Evaluation

Cognitive performance was assessed through two behavioural tests widely used to evaluate hippocampal function: the Y-maze for spatial recognition memory [21,29] and the NOR test [20,30]. Both tasks were selected for their simplicity, reproducibility, and low stress level, making them suitable for longitudinal or methodological studies in rodents [31].

#### 2.4.1. Y-Maze Test

**Figure 3:**
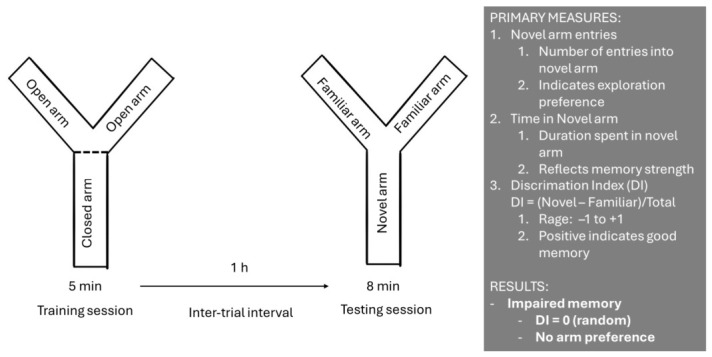
Schematic representation of the experimental procedure for spatial recognition memory testing using the Y-maze. This was a three-part experiment where we first closed the designated novel arm and left the rat to explore the familiar arms. After an inter-trial interval of one hour, the rats were reintroduced to the maze and left to explore.

We used the Y-maze test to evaluate spatial recognition memory and short-term memory acquisition. A schematic representation of the experimental procedure is shown in Figure 3. Testing was carried out using a Y-shaped maze with three light-coloured, opaque arms arranged at 120° angles. An entry was considered when all four limbs of the rat were within an arm’s length. The test consisted of three phases [21]:

(1) Training session—a designated arm was closed, and the rat was placed in one of the open arms for 5 min to explore.
(2) Inter-trial interval—the rat was removed from the maze for 1 h.
(3) Testing session—the designated closed arm was considered the novel arm and opened for exploration. The rat was placed again in a familiar arm and left to explore for 8 min.

Time spent and the number of entries into the newly opened arm were recorded and used to calculate discrimination indices [21,32]. Each session was video-recorded and later analyzed by an observer blinded to the experimental conditions [21,32].

The time-based discrimination index (DI_time) was calculated as follows:

DI_time = (T_novel − T_familiar)/T_total,(1)

where T_novel denotes the time spent in the novel arm and T_familiar denotes the time spent in the familiar arms.

#### 2.4.2. NOR Test

Working memory for short- and long-term recognition was assessed using the NOR test.

**Figure 4:**
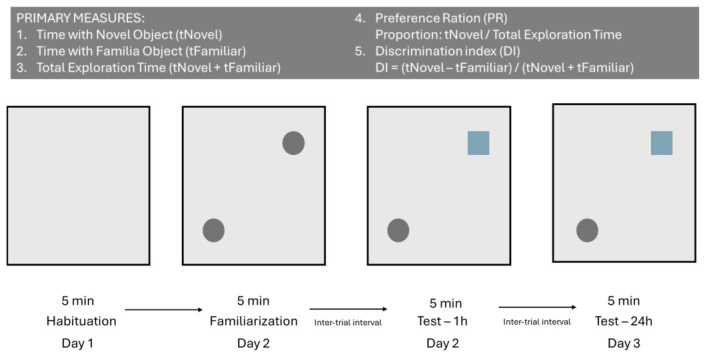
Schematic representation of the experimental procedure for the NOR test maze with evaluations for the 3-day session. The first day was used for habituation with the box empty, solely for the animals to explore. On the second day, the animals were introduced to two familiar objects and left for 5 min for exploration. After the inter-trial interval, the test began. Short-term recognition memory was evaluated at 1 h, whereas long-term recognition memory was evaluated at 24 h.

The apparatus for the maze consisted of a square open field arena constructed of black PVC plastic (70 cm × 70 cm × 60 cm). The objects used were two flasks for familiarization and a plastic block-shaped object for a novel object. The objects were placed in the opposite corners of the arena (NE and SW), approximately 2 cm from the wall. A 5 min test was conducted in all experimental procedures: habituation, familiarization, 1 h testing, and 24 h testing. Exploration was defined as sniffing or touching the object with the nose within a 2 cm distance [20,30,33]. Figure 4 summarizes the protocol used for the NOR test.

The discrimination index (DI) was calculated as follows:

DI = (t_novel − t_familiar)/(t_novel + t_familiar), (2)

where t_novel represents the time spent exploring the novel object and t_familiar represents the time spent exploring the familiar object.

The 1 h and 24 h retention tests were performed on the same animals and therefore represent repeated measures within subjects. These points were analyzed separately to describe short-term and longer-term recognition performance.

All behavioural sessions were conducted during the light phase of the cycle, in a quiet and controlled environment, to minimize external distractions. Between each trial, the apparatus and objects were cleaned with 70% ethanol to remove any olfactory cues.

### 2.5. Histology

At the end of the experiment, euthanasia was performed by administering an overdose of an anesthetic (Isoflutek, 1000 mg/g, Karizoo, Barcelona, Spain), ensuring rapid death without physical or psychological suffering. All procedures were carried out in designated necropsy rooms separated from the animal housing areas, following standard veterinary and ethical requirements.

Brains were carefully removed and post-fixed in 10% neutral buffered formalin for 24 h. Tissue processing was carried out using an Excelsior™ AS Tissue Processor (Epredia Holdings Ltd., Portsmouth, NH, USA) according to a standardized protocol. The sequence included graded ethanol dehydration (99.9%, Laboratorium Life Science, Galați, Romania), clearing in xylene (Epredia Holdings Ltd.), and paraffin infiltration (Histoplast PE, Epredia Holdings Ltd.). Subsequently, the samples were embedded in paraffin blocks (Histoplast PE) and sectioned at a thickness of 5 μm using a semi-automatic microtome (CUT 5062, SLEE medical GmbH, Nieder-Olm, Germany). The sections were then stained with H&E, employing Mayer’s hematoxylin and 0.5% aqueous eosin Y (both from Sigma-Aldrich, Burlington, VT, USA) and Congo red (Epredia Holdings Ltd.). H&E staining allowed for assessment of the hippocampal tissue’s overall structure, including neuronal arrangement, cell density, and any degenerative changes. Congo red staining was performed on brain sections, which were subsequently examined under both brightfield and polarized light microscopy to assess birefringence.

### 2.6. Statistical Analysis

Data are presented as mean ± SEM. Statistical analyses included one-sample Student’s *t*-tests, independent-samples Student’s *t*-tests, repeated-measures ANOVA, and Mann–Whitney U tests, as appropriate.

Given the small sample size (n = 6 per group), formal testing of normality (e.g., Shapiro–Wilk test) and sphericity was not considered sufficiently reliable for inferential use. Instead, data distributions were examined descriptively.

Parametric tests were retained for consistency with commonly used analytical approaches in similar experimental studies; however, all key between-group comparisons were additionally supported by non-parametric analyses (Mann–Whitney U test).

Repeated-measures ANOVA was applied to explore within-subject patterns of arm exploration. However, given the inability to meaningfully assess sphericity at this sample size, these results are interpreted as exploratory.

No formal a priori power calculation was performed. The sample size was selected for this feasibility-oriented study and should therefore be considered exploratory.

Effect sizes are reported together with their 95% confidence intervals to reflect both the magnitude and uncertainty of the observed effects.

All analyses were performed using JASP (version 0.95.4.0), and *p* < 0.05 was considered statistically significant.

## 3. Results

### 3.1. Morphological Confirmation of Aβ_1–42_ Fibril Formation

**Figure 5:**
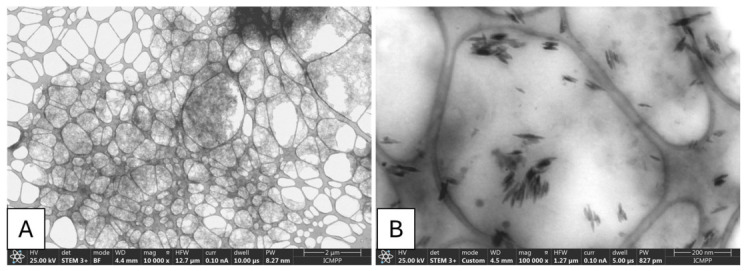
STEM ultrastructural characterization of Aβ_1–42_ aggregates. (**A**) Low-magnification STEM image (10,000×) showing an extensive network of fibrillar Aβ_1–42_ aggregates formed after incubation at 37 °C for 7 days. (**B**) High-magnification STEM image (100,000×) illustrating elongated amyloid fibrils and fibrillar bundles, consistent with mature insoluble amyloid assemblies prior to in vivo administration.

STEM analysis revealed abundant fibrillar structures consistent with insoluble Aβ_1–42_ aggregates. The peptide assemblies appeared as elongated, dense fibrils with variable length and thickness, forming intertwined networks and compact structures. These ultrastructural features are consistent with advanced amyloid fibril maturation, supporting the suitability of the preparation for in vivo administration in amyloid-based neurodegenerative models (Figure 5).

### 3.2. Induction of AD

We performed stereotactic bilateral injection of pre-aggregated Aβ_1–42_ peptide into the hippocampus in all six experimental animals from the AD group. The surgical procedure was well-tolerated, with no perioperative or postoperative complications or unexpected mortality. After the intervention, all animals were monitored and confirmed to have normal feeding behaviour and spontaneous mobility.

Targeting accuracy was ensured by using the stereotaxis apparatus for both cranial drilling and peptide injection. The slow infusion rate and the five-minute retention of the Hamilton syringe at the injection site minimized reflux and ensured adequate diffusion of the amyloid suspension. The overall procedure duration averaged 30–35 min per animal. The protocol was technically feasible and suitable for further use.

### 3.3. Cognitive Evaluation

#### 3.3.1. Y-Maze Test

**Figure 6:**
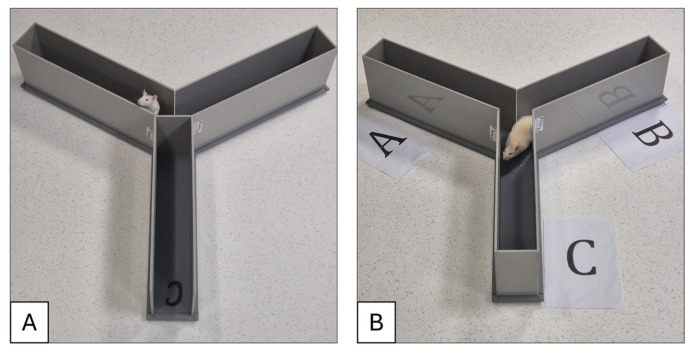
Y-maze evaluation in (**A**) the training session with the designated novel arm closed and the animal exploring the familiar arms, and (**B**) the testing session with all three arms opened for exploration.

The Y-maze test was performed one week after amyloid peptide injection on both wild-type (n = 6) and amyloid-injected rats (n = 6). The designated novel arm was arm C. We evaluated spatial recognition memory using the Y-maze with a blocked arm, as described in Figure 6.

Rats injected with Aβ_1–42_ exhibited a reduced tendency to enter and explore the novel arm compared with control animals. Aβ-injected rats showed fewer transitions, with a notable preference for one previously visited arm and limited exploration of the third arm.

In the control group (WT), the time-based discrimination index (DI_time) was significantly greater than zero (0.135 ± 0.104; one-sample *t*-test: *t*(5) = 3.196, *p* = 0.024), indicating preserved novelty preference in this task. The AD group showed a negative DI_time (−0.179 ± 0.203), which did not differ significantly from zero (*t*(5) = −2.152, *p* = 0.084), suggesting reduced novelty preference. The analysis of the one-sample Student’s *t*-test is shown in Table 2.

**Figure 7:**
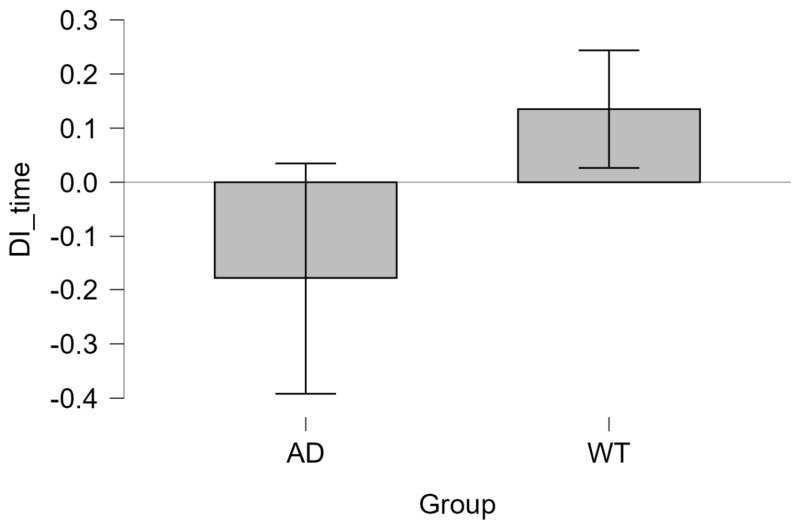
Y-maze performance assessed by time-based discrimination index (DI_time). WT animals show a positive DI_time, indicating preference for the novel arm, whereas AD animals exhibit reduced or negative DI_time values, consistent with reduced novelty preference in the blocked-arm Y-maze task. Data are presented as mean ± SEM; the horizontal line denotes chance level (DI = 0).

DI_time was lower in the AD group compared with WT controls (independent samples *t*-test: *t*(10) = −3.368, *p* = 0.007), with a measured effect size of Cohen’s d = −1.945. These results are consistent with reduced spatial recognition performance in the Aβ_1–42_ group, although they should be interpreted cautiously given the small sample size and the absence of sham-operated controls. The relatively large variability observed within the AD group further limits the strength of this finding (Table 3 and Figure 7).

The entry-based discrimination index (DI_entries) showed that the AD group scored lower than the WT group. This gap, however, did not reach statistical significance (*t*(10) = −1.898, *p* = 0.087). The higher variability observed in entry counts may have contributed to this result.

Exploration behaviour was analyzed through a repeated-measures ANOVA, using arm type (novel, familiar 1, familiar 2) as the within-subject variable, while grouping animals into WT and AD formed the between-subject contrast (Table 3). A significant ARM X GROUP interaction was observed (F(2,20) = 5.829, *p* = 0.010), suggesting that WT and AD animals distributed their exploration time differently across maze arms. Data supporting these points appear in Table 4.

Given the limited sample size, assumptions underlying repeated-measures ANOVA could not be fully verified, and these findings should be interpreted with caution. Overall, these observations suggest a reduced spatial recognition performance in the Aβ_1–42_ group.

#### 3.3.2. Novel Object Recognition (NOR) Test

**Figure 8:**
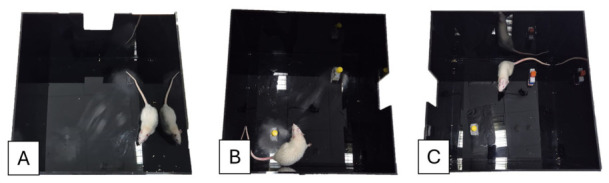
NOR test maze evaluation for the three-day experiment. (**A**) Habituation period where rats were left for 5 min in the empty box to explore. (**B**) Training period where the rats were introduced to two identical objects and left for 5 min to explore. (**C**) Testing period performed at 1 h and 24 h after the training. One of the familiar objects was changed to a novel one, and animals were recorded.

The NOR test was performed two weeks after the Aβ_1–42_ injection. The habituation and training sessions showed normal adaptation and exploratory behaviour with intact mobility. Figure 8 illustrates the experimental protocol. During the testing phase, at 1 h and 24 h, amyloid-induced rats showed a lower discrimination index compared to the control group. The injected animals explored both the familiar and novel objects for similar durations, suggesting reduced recognition memory. In contrast, control animals consistently spent more time exploring the novel object, reflecting preserved cognitive performance.

The results for both the 1 h and 24 h tests are shown in Table 5. At the 1-h retention interval (DI_1h), the discrimination index was significantly lower in the Aβ group compared to WT animals (*t*(8) = −3.08, *p* = 0.015). This difference was also supported by the Mann–Whitney U test (U = 2, *p* = 0.032). WT animals showed positive DI values, whereas Aβ-treated animals displayed reduced or negative discrimination indices.

At the 24 h retention interval (DI_24h), group differences were also observed, with lower DI values in the Aβ group (*t*(8) = −3.48, *p* = 0.008). This result was confirmed by the Mann–Whitney U test (U = 0, *p* = 0.008). WT animals maintained positive discrimination indices, while Aβ-treated animals showed a reduced preference for the novel object.

Statistical analysis showed a large effect size, but given the small sample size, these results should be interpreted with caution. Overall, the findings indicate reduced object recognition performance in the Aβ group.

### 3.4. Histology

**Figure 9:**
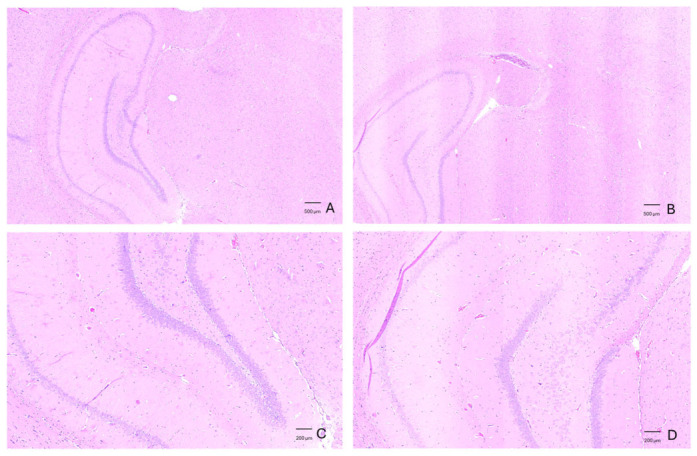
Histological analysis with H&E-stained hippocampal sections from control and Aβ_1–42_-injected rats. (**A**) Control (WT) hippocampus at low magnification showing preserved overall architecture and well-defined laminar organization. (**B**) Aβ_1–42_-injected hippocampus at low magnification, with subtle structural alterations and focal areas of tissue disorganization. (**C**) Higher magnification of the control hippocampus, illustrating compact and clearly delineated neuronal layers. (**D**) Higher magnification of Aβ_1–42_-injected hippocampus showing a more diffuse cellular arrangement and partial disruption of laminar organization.

Histological evaluation using hematoxylin and eosin (H&E) staining showed a preserved hippocampal architecture in control animals, characterized by well-defined and compact neuronal layers. The pyramidal and granular cell layers exhibited normal organization, with clearly delineated laminar boundaries (Figure 9A,C). In contrast, sections obtained from Aβ_1–42_-injected animals showed subtle structural alterations, including a less compact laminar organization and focal areas of tissue disorganization within the hippocampal formation. At higher magnification, these changes were reflected by a more diffuse cellular distribution and partial blurring of the normal laminar pattern compared to controls (Figure 9B,D). While these findings are consistent with tissue-level changes following peptide administration, the absence of a sham-operated group does not allow for complete exclusion of procedure-related effects.

**Figure 10:**
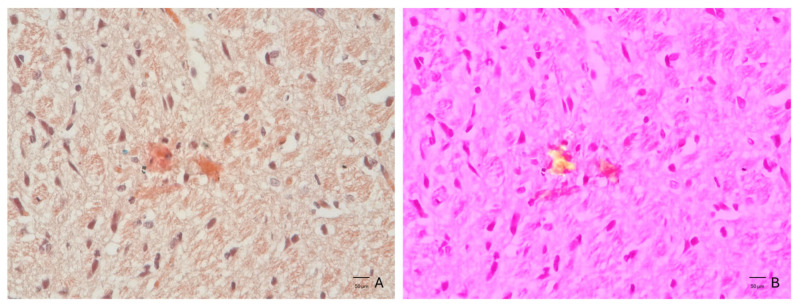
Congo red staining and polarized light microscopy of hippocampal sections. (**A**) Representative brightfield image showing Congo red-positive deposits appearing as red–orange structures within the tissue. (**B**) The corresponding polarized light image (right) exhibited birefringence of these deposits, consistent with an organized fibrillar arrangement. These features were observed in Aβ_1–42_-injected animals, while control sections did not show comparable birefringent structures.

Congo red staining revealed focal red–orange deposits within the examined sections of Aβ_1–42_-injected animals (Figure 10A). Under polarized light, these deposits displayed birefringence consistent with amyloid-like congophilic material, in line with the characteristic apple-green appearance described in the literature, with minor variations in hue likely related to imaging conditions (Figure 10B). Control sections did not display comparable birefringent structures.

## 4. Discussion

A major challenge in preclinical Alzheimer’s disease research is the substantial methodological heterogeneity across experimental models, which compromises reproducibility and translational potential. Methodological differences in protocols for amyloid peptide preparation, injected dose, stereotactic coordinates and behavioural validation strategies have been repeatedly highlighted in narrative reviews as a critical source of inconsistency [5,8]. As summarized in Table 1, there is considerable heterogeneity across studies in terms of injection parameters, including volume, concentration, aggregation state, and stereotactic targeting. Importantly, even when similar injection volumes are used, differences in peptide concentration result in substantial variability in the effective delivered dose, which may contribute to differences in behavioural and histological outcomes.

In this context, the present study explores the feasibility of an intrahippocampal Aβ_1–42_ administration framework. The primary aim was to provide a framework and an ethically compliant experimental design for intrahippocampal Aβ_1–42_ administration in rats.

Our findings show that bilateral stereotactic delivery of aggregated Aβ_1–42_ into the hippocampus is associated with reduced hippocampus-dependent cognitive performance accompanied by localized histopathological changes observed under the present experimental conditions. The surgical procedure was well-tolerated, with no perioperative complications, supporting its feasibility for repeated experimental use. Precise stereotactic targeting and controlled infusion parameters contributed to consistent peptide delivery, addressing a key source of the variability reported in previous studies.

The stereotactic coordinates used in this study were selected to target the dorsal hippocampus, particularly the CA1 region. This choice was guided by the well-established involvement of the dorsal hippocampus in spatial learning and recognition memory [34,35], which are the primary domains assessed by the Y-maze and novel object recognition tests [21]. In contrast, ventral hippocampal regions are more closely associated with emotional and affective processing, which were not directly evaluated in the present study. Therefore, targeting the dorsal CA1 region provides a functionally relevant approach for investigating behavioural alterations in this experimental context.

The extended aggregation period (7 days at 37 °C) favours the formation of mature fibrillar Aβ species, which are associated with extracellular fibrillar aggregates and Congo red positivity. In contrast, shorter incubation protocols typically generate oligomeric forms that are more closely linked to synaptic dysfunction. Therefore, the present preparation likely favours fibrillar aggregate formation, which should be taken into account when interpreting the observed behavioural and histological findings.

Behavioural assessment using the Y-maze and the NOR test revealed consistent changes in hippocampus-dependent behavioural performance in the Aβ_1–42_-injected animals. The amyloid-induced group exhibited reduced novelty preference and altered exploration patterns in the Y-maze, with lower discrimination indices in both short-term and long-term object recognition in the NOR test. Although group differences were observed across both maze explorations, these findings should be interpreted with caution, given the limited sample size. While relatively large effect sizes were noted, their confidence intervals were wide, reflecting a degree of uncertainty in the estimates. Importantly, total exploratory activity did not differ significantly between groups, indicating that the observed behavioural differences were unlikely to be explained by major changes in locomotor activity [21,29,30,32,33,36].

Congo red staining represents one of the most widely used histochemical techniques for detecting amyloid deposits in biological tissue [37]. The combination of polarized light microscopy with Congo red staining provides supportive, but not definitive, evidence of amyloid-like congophilic material. In the analyzed sections, extracellular aggregates with red–orange coloration were identified within the brain parenchyma using brightfield microscopy and Congo red staining. These aggregates exhibit morphological features consistent with congophilic fibrillar material, including irregular shapes and localization within the extracellular neuropil. The presence of birefringent structures under polarized light provides a reliable method for detecting amyloid-like fibrillar structures. However, while these findings support the presence of amyloid-like congophilic material, the absence of quantitative histological analysis (e.g., Congo red-positive area or plaque density) limits the ability to objectively assess the extent of these Congo red-positive findings. Therefore, these observations should be interpreted as a qualitative assessment rather than quantitative validation, and future studies should incorporate quantitative or semi-quantitative approaches to strengthen histological assessment.

Taken together, our findings support the feasibility and internal consistency of intrahippocampal Aβ_1–42_ administration within a controlled experimental setting. The experiment is technically accessible and ethically compliant, allowing for its use in similar laboratory contexts.

However, as this study is based on a single experimental cohort, it does not allow for firm conclusions regarding reproducibility. Demonstrating reproducibility would require independent validation and direct comparison with other established protocols.

Several limitations should be acknowledged. The experimental timeline was limited to four weeks, capturing early pathological changes without addressing long-term disease progression. In addition, the small sample size may limit statistical power.

An important limitation of the present study is the absence of a sham-operated control group. As a result, we cannot fully exclude the contribution of surgical factors, including stereotactic needle insertion, anesthesia, and procedure-related neuroinflammatory responses, to the observed behavioural and histological changes. An additional limitation is the absence of systematic blinding across all experimental stages, as well as the lack of formal randomization in group allocation, both of which may introduce potential sources of bias in behavioural and histological assessments.

Furthermore, in the absence of histological data from sham-operated animals, it is not possible to determine the extent to which the observed structural alterations are specifically related to Aβ_1–42_ administration versus procedure-induced tissue effects. Consequently, the relationship between histological findings and behavioural performance should be interpreted with caution. An additional limitation is the absence of a formal a priori sample size calculation. With six animals per group, the present study was primarily suited to detect large effects, whereas smaller or moderate differences may have remained undetected.

Therefore, the observed behavioural and histological findings should be interpreted as reflecting the combined effects of peptide administration and procedural factors rather than exclusively Aβ-specific pathology. Future studies incorporating sham-operated controls will be essential to isolate peptide-specific effects and to further validate the model.

## 5. Conclusions

This study presents a feasible and technically consistent framework for establishing an intrahippocampal Aβ_1–42_ rat model, demonstrating behavioural changes and histological alterations under the present experimental conditions. However, further validation, including the use of sham-operated controls, will be necessary to confirm the specificity of these findings for Aβ-related pathology. Histological analysis using standard stains provides supportive evidence of amyloid-like features, although additional molecular validation would be required for definitive characterization.

This approach provides a technically accessible and cost-effective framework for intrahippocampal Aβ_1–42_ administration in rats, producing consistent behavioural and histological observations within the present experimental setting. By detailing each procedural step, the study offers a structured methodological basis that may support future replication and comparative studies.

However, under the current experimental conditions, the model should be interpreted as a feasibility-oriented framework reflecting the combined effects of peptide administration and procedural factors, rather than a fully validated Aβ-specific disease model. Further studies incorporating appropriate controls and molecular characterization will be required to clarify the specificity of the observed findings.

## Figures and Tables

**Table 1:** Comparative overview of intrahippocampal Aβ_1–42_ protocols. The table summarizes representative studies using intrahippocampal Aβ_1–42_ administration, highlighting variability in injection volume, peptide concentration, aggregation state, behavioural assessment timelines, histological validation methods, stereotactic coordinates, and sample size.

Author, Year, Reference	Volume (μL per Side)	Concentration (μg/μL)	Aggregation	Behavioural Timeline (Days After Injection)	Histology	Coordinates (mm)	Sample Size (n/Group)
Karthick et al., 2019 [11]	1	1	Oligomeric	30	Congo red (+)	AP −4.0 ML +/−3.0 DV −3.6	4
Scuderi C. et al., 2014 [12]	2.5	2	Fibrillar	18	GFAP (+)	AP −3.00 ML +/−2.2 DV −2.8	9–12
Facchinett R. et al., 2018 [13]	2.5	2	Oligomeric	Not reported	Not reported	AP −3.00 ML +/−2.2 DV −2.8	Not applicable
Karimi-Sales et al., 2020 [14]	3	0.01	Fibrillar	19	Not reported	CA1 region	14
Alimohammadi-Kamalabadi et al., 2016 [15]	4	0.5	Oligomeric	42	Thioflavin-T (+)	AP −3.9 ML +/−2.2 DV −2.7	8
Bai W et al., 2016 [16]	5	1	Oligomeric	14	Not reported	AP −3.2 ML +/−2.5 DV −3.5	4
Hidisoglu et al., 2022 [17]	5	0.01 0.1 1	Oligomeric	Not reported	Not reported	AP −3.24 ML +/−2.5 DV −3.5	8
Shakerin et al., 2020 [18]	5	1	Fibrillar	21	Congo red (+)	AP −3.36 ML +/−1.6 DV −3.2	7
Babei et al., 2023 [19]	5	1	Oligomeric	12	Not reported	Not reported	8

**Table 2:** One-sample Student’s *t*-test for Y-maze DI_time. DI_time values were compared against the chance level (0) in the WT and AD groups. Positive values indicate preference for the novel arm. WT animals showed a DI_time of significantly above zero, whereas AD animals did not differ significantly from chance.

	*t*	df	*p*	Mean Difference
DI_time_WT	3.196	5	0.024	0.135
DI_time_AD	−2.152	5	0.084	−0.179

Note. For the Student *t*-test, the alternative hypothesis specifies that the mean is different from 0. Note. Student’s *t*-test.

**Table 3:** Independent samples *t*-test for Y-maze DI_time. DI_time was significantly lower in AD animals compared with WT controls, with a measured effect size reported together with its 95% confidence interval (Cohen’s d).

						95% CI for Cohen’s d	
	*t*	df	*p*	Cohen’s d	SE Cohen’s d	Lower	Upper
DI_time	−3.368	10	0.007	−1.945	0.805	−3.324	−0.504

Note. Student’s *t*-test.

**Table 4:** Repeated-measures ANOVA of Y-maze arm exploration. The analysis revealed a significant interaction between maze arm (novel, familiar 1, familiar 2) and experimental group, indicating that WT and AD animals distributed their exploration time differently across arms.

Cases	Sum of Squares	df	Mean Square	F	*p*
ARM (1)	2291	2	1145.4	3.047	0.070
ARM (1) ✻ Group	4382	2	2191.0	5.829	0.010
Residuals	7518	20	375.9		

Note. Type III Sum of Squares.

**Table 5:** Independent samples comparisons for novel object recognition performance. Group differences in discrimination index (DI) at 1 h and 24 h retention intervals were assessed using Student’s *t*-test and confirmed with the non-parametric Mann–Whitney U test.

Independent Samples *t*-Test					
	Test	Statistic	*p*	Effect Size	SE Effect Size
DI_1h	Student	−3.081	0.015	−1.949	0.883
	Mann–Whitney	2.000	0.032	0.840	0.365
DI_24h	Student	−3.479	0.008	−2.201	0.940
	Mann–Whitney	0.000	0.008	1.000	0.365

Note. For the Student *t*-test, the effect size is given by Cohen’s d. For the Mann–Whitney test, the effect size is given by the rank biserial correlation.

## Data Availability

The original contributions presented in this study are included in the article. Further inquiries can be directed to the corresponding author.
